# SERPINB1 expression is predictive for sensitivity and outcome of cisplatin-based chemotherapy in melanoma

**DOI:** 10.18632/oncotarget.6956

**Published:** 2016-01-20

**Authors:** Christoph Willmes, Rajiv Kumar, Jürgen C. Becker, Isabella Fried, P. Sivaramakrishna Rachakonda, Lidia M. Poppe, Sonja Hesbacher, Dirk Schadendorf, Antje Sucker, David Schrama, Selma Ugurel

**Affiliations:** ^1^ Department of Dermatology, University Hospital Würzburg, Würzburg, Germany; ^2^ Division of Molecular Genetic Epidemiology, German Cancer Research Center, Heidelberg, Germany; ^3^ Translational Skin Cancer Research, Deutsches Konsortium für Translationale Krebsforschung (DKTK), Essen, Germany; ^4^ Department of Dermatology, University Duisburg-Essen, Essen, Germany; ^5^ Department of Dermatology, Medical University of Graz, Graz, Austria

**Keywords:** melanoma, SERPINB1, cisplatin, chemotherapy, predictive marker

## Abstract

Despite of highly effective new therapeutic strategies, chemotherapy still is an important treatment option in metastatic melanoma. Since predictors of chemotherapy response are rare, drugs and regimens are currently chosen arbitrarily. The present study was aimed at the identification of molecular markers predicting the outcome of chemotherapy in melanoma. Tumor biopsies from metastatic lesions were collected from 203 stage IV melanoma patients prior to chemotherapy onset and used for gene expression profiling (*n* = 6; marker identification set), quantitative real-time PCR (*n* = 127; validation set 1), and immunohistochemistry on tissue microarrays (*n* = 70; validation set 2). The results were correlated to the tumors' *in-vitro* chemosensitivity and to the patients' *in-vivo* chemotherapy outcome. SERPINB1 was found to correlate to the *in-vitro* sensitivity to cisplatin-containing chemotherapy regimens (*p* = 0.005). High SERPINB1 gene expression was associated with favorable tumor response (*p* = 0.012) and prolonged survival (*p* = 0.081) under cisplatin-based chemotherapy. High SERPINB1 protein expression in tumor tissue from cisplatin-treated patients was associated with a favorable survival (*p* = 0.011), and proved as an independent predictor of survival (*p* = 0.008) by multivariate analysis. We conclude, that SERPINB1 expression, although not functionally involved, is predictive for the outcome of cisplatin-based chemotherapy in melanoma, and thus may be useful to personalize melanoma chemotherapy.

## INTRODUCTION

Therapy of metastatic melanoma is currently undergoing a rapid and radical structural change. This is due to two newly developed groups of therapeutics, inhibitors attacking the mitogen-activated protein (MAP) kinase pathway like vemurafenib, dabrafenib, and trametinib, and immune checkpoint blockers like ipilimumab, nivolumab and pembrolizumab. Agents from both groups were able to demonstrate a prolongation in overall survival of melanoma patients in randomized phase-3 trials [1, 2], a result which has never been successfully shown before for any systemic therapeutic. Before this new era of targeted agents, the standard treatment of metastatic melanoma was chemotherapy. Herein, monochemotherapy with dacarbazine (DTIC) served as standard first-line treatment, while combination chemotherapy, mainly with cisplatin-based regimens, was used in second-line therapy. After the astonishing results of the new drugs, it was assumed that the era of chemotherapy in melanoma was over. Nevertheless, chemotherapy of melanoma is not outdated; it still plays an important role, but in other patient settings than before [3, 4]. This is particularly in patients not harbouring a druggable MAP kinase pathway mutation, or in patients who are mutation-carriers but are not or no longer responding to the respective targeted agents. Moreover, chemotherapy is indicated in patients who are not suitable or not responding to immune checkpoint blockers. Thus, chemotherapy was switched from a primarily first-line to a mainly second- or higher line treatment strategy. However, this renders it not less important for the standard of care of metastatic melanoma, and still most melanoma patients with distant metastasis receive one or more lines of chemotherapy sooner or later during their course of disease.

Thus, to improve the outcome of chemotherapy in melanoma it is of high importance to stratify patients into both, groups of high or low probability to benefit from chemotherapy, and subgroups of selected chemotherapy agents or combination regimens for patients with high probability to respond. Such a personalized treatment strategy would be of particular importance since chemotherapy has up to now been shown repeatedly not to prolong patient survival if applied in an unselective mode [5, 6]. The new inhibitors of the MAP kinase pathway imply the advantage, that the patient population with a high probability to respond can be easily identified by testing the tumor tissue for the druggable mutation. Thus, the presence of these mutations can be simultaneously used as drug target and as biomarker of therapy response [7, 8]. For chemotherapy, in contrast, predictive markers helping to stratify patients for specific drugs or regimens are not known. Thus, the choice of mono- or combination chemotherapeutics for melanoma is therefore currently made arbitrarily. We recently demonstrated in a phase-2 trial, that the *in vitro* chemosensitivity profile determined from fresh tumor tissue can be used for the stratification of melanoma patients for different groups of chemotherapeutic regimens [9]. Moreover, in those patients who were subsequently treated with a sensitivity-directed chemotherapy, the respective chemosensitivity measured for each tumor correlated to treatment response and patient survival [9].

In the present study we aimed at the identification of molecular markers predicting the outcome of chemotherapy in metastatic melanoma. For this purpose, we first performed a gene expression profiling of melanoma cell lines established from tumor tissue biopsies taken before the onset of chemotherapy, in order to identify genes which are differentially expressed in tumor cells from chemotherapy responders compared to non-responders. From the resulting list of differentially expressed genes, five candidates were chosen for further validation. For this purpose, we analyzed tumor cells isolated from tissue biopsies of metastatic melanoma lesions for their *in vitro* chemosensivity towards a panel of chemotherapeutics as single agents or combinations. Parts of these tumor tissue biopsies were used to analyze the expression of the candidate genes in two independent validation sets, either on transcriptional level in cryopreserved tissue samples (validation set 1), or on protein level by tissue microarray immunohistochemistry analysis of formalin-fixed paraffin-embedded samples (validation set 2). The findings resulting from these experiments were correlated to the *in vitro* chemosensitivity of the corresponding tumors, as well as to the clinical outcome of the first subsequent therapy in the corresponding patients.

## RESULTS

### Patient characteristics

**Figure 1:**
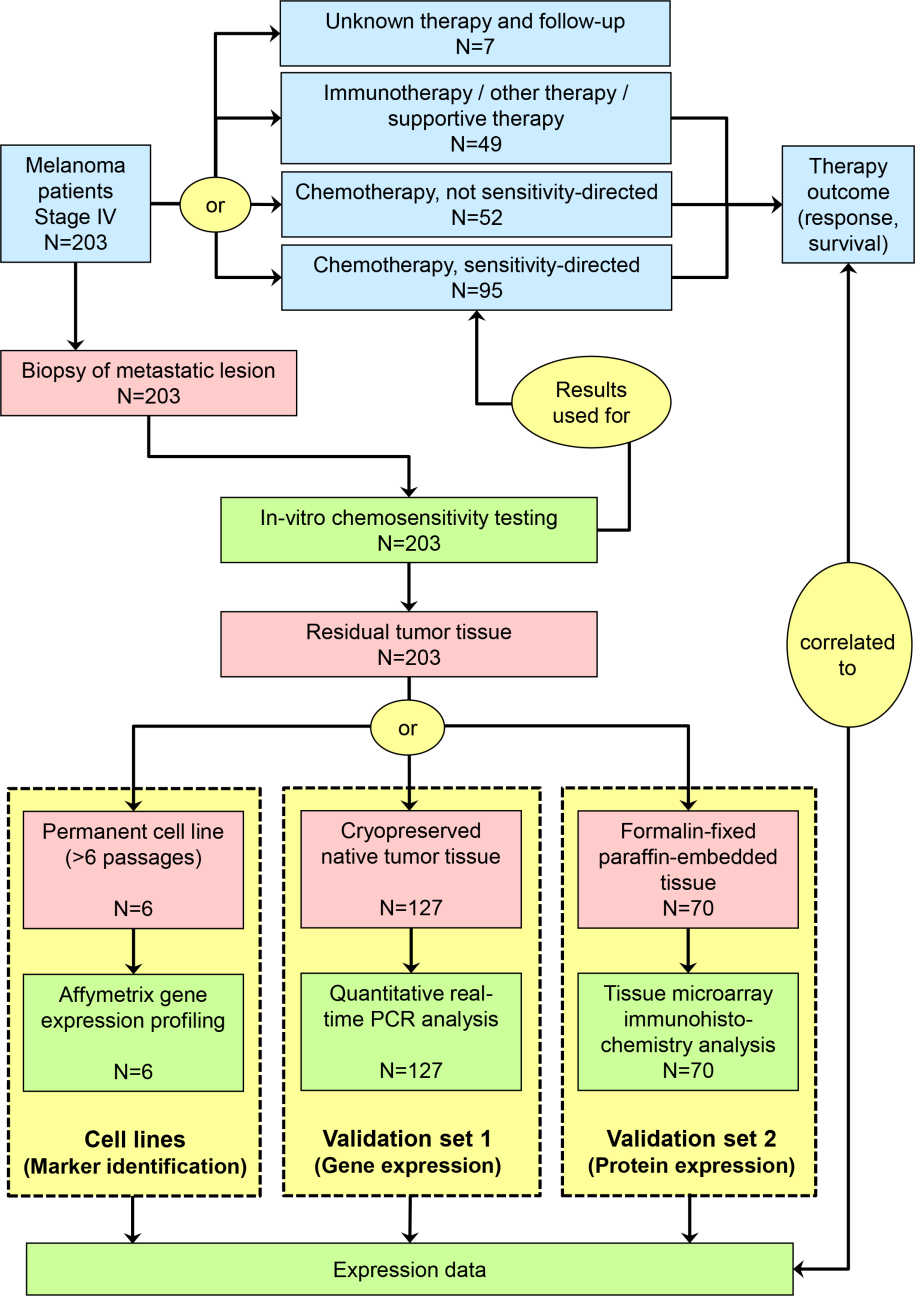
Schematic presentation of study flow Red symbols represent biomaterials, blue symbols represent clinical procedures and results, and green symbols represent experimental procedures and results.

203 stage IV melanoma patients were subject of chemosensivity testing and subsequent workup of banked biomaterials throughout this study. This total population consisted of three independent sets of patients: six patients were investigated based on cell line materials (marker identification set), 127 patients were investigated based on cryopreserved tissue materials (validation set 1), and 70 patients were analyzed based on FFPE tissue samples (validation set 2) (Table 1, Figure 1). 62 patients from validation set 1, and 34 patients from validation set 2 participated in clinical multicenter trials of sensitivity-directed chemotherapy ([9]; ClinicalTrials.gov: NCT00779714).

**Table 1:** Patient characteristics

		Validation set 1 127 (100.0%)		Validation set 2 70 (100.0%)	
**Gender**	male	77	(60.6%)	44	(62.8%)
	female	50	(39.4%)	26	(37.1%)
**Median age/years (range)**		61.6	(16.4–91.2)	62.3	(25.9–90.8)
**Localization of primary**	skin	87	(68.5%)	53	(75.7%)
	mucosa	8	(6.3%)	3	(4.3%)
	uvea	2	(1.6%)	3	(4.3%)
	occult	14	(11.0%)	8	(11.4%)
	unknown	16	(12.6%)	3	(4.3%)
**M category (AJCC)**	M1a	12	(9.4%)	5	(7.1%)
	M1b	18	(14.2%)	6	(8.6%)
	M1c	82	(64.6%)	55	(78.6%)
	unknown	15	(11.8%)	4	(5.7%)
**Serum LDH**	≤ UNL	43	(33.9%)	19	(27.1%)
	> UNL	69	(54.3%)	47	(67.2%)
	unknown	15	(11.8%)	4	(5.7%)
**ECOG performance status**	0	43	(33.9%)	46	(65.7%)
	1	34	(26.8%)	22	(31.4%)
	≥ 2	19	(15.0%)	2	(2.9%)
	unknown	31	(24.3%)	0	(0.0%)
**Metastatic site biopsied for chemosensitivity testing**	skin/subcutaneous	63	(49.6%)	50	(71.4%)
	lymph node	51	(40.2%)	15	(21.4%)
	visceral organ	13	(10.2%)	5	(7.1%)
**Best chemosensitivity index**	median/mean (range)	131/136	(1–360)	150/154	(7–315)
	≤ 100	44	(34.6%)	17	(24.3%)
	> 100	83	(65.4%)	53	(75.7%)
**First therapy after chemosensitivity testing^1^**	chemotherapy	80	(63.0%)	61	(87.1%)
	sensitivity-directed	52	(40.9%)	37	(52.9%)
	cisplatin + paclitaxel	18	(14.2%)	22	(31.4%)
	cisplatin + gemcitabine	9	(7.1%)	0	(0.0%)
	treosulfan + gemcitabine	25	(19.8%)	15	(21.4%)
	not sensitivity-directed	28	(22.0%)	24	(34.3%)
	dacarbazine (DTIC)	20	(15.7%)	24	(34.3%)
	other chemo regimen	8	(6.3%)	0	(0.0%)
	immunotherapy	15	(11.8%)	3	(4.3%)
	other/supportive therapy	25	(19.7%)	6	(8.6%)
	unknown	7	(5.5%)	0	(0.0%)
**SERPINB1 relative gene expression^2^**	median/mean (range)	0.88/1.08	(0.04–4.61)	n.d.	
**SERPINB1 protein expression score^2^**	median/mean (range)	n.d.	4.0/3.7	(0–5)	

1 First systemic treatment given to the patient after the procedure of chemosensitivity testing

2 SERPINB1 expression as detected in tissue samples obtained for chemosensitivity testing (for details see Patients and Methods)

Abbreviations: AJCC, American Joint Committee on Cancer; LDH, lactate dehydrogenase; UNL, upper normal limit; ECOG, Eastern Cooperative Oncology Group

### Differentially expressed genes in chemosensitive versus chemoresistant tumors

**Figure 2:**
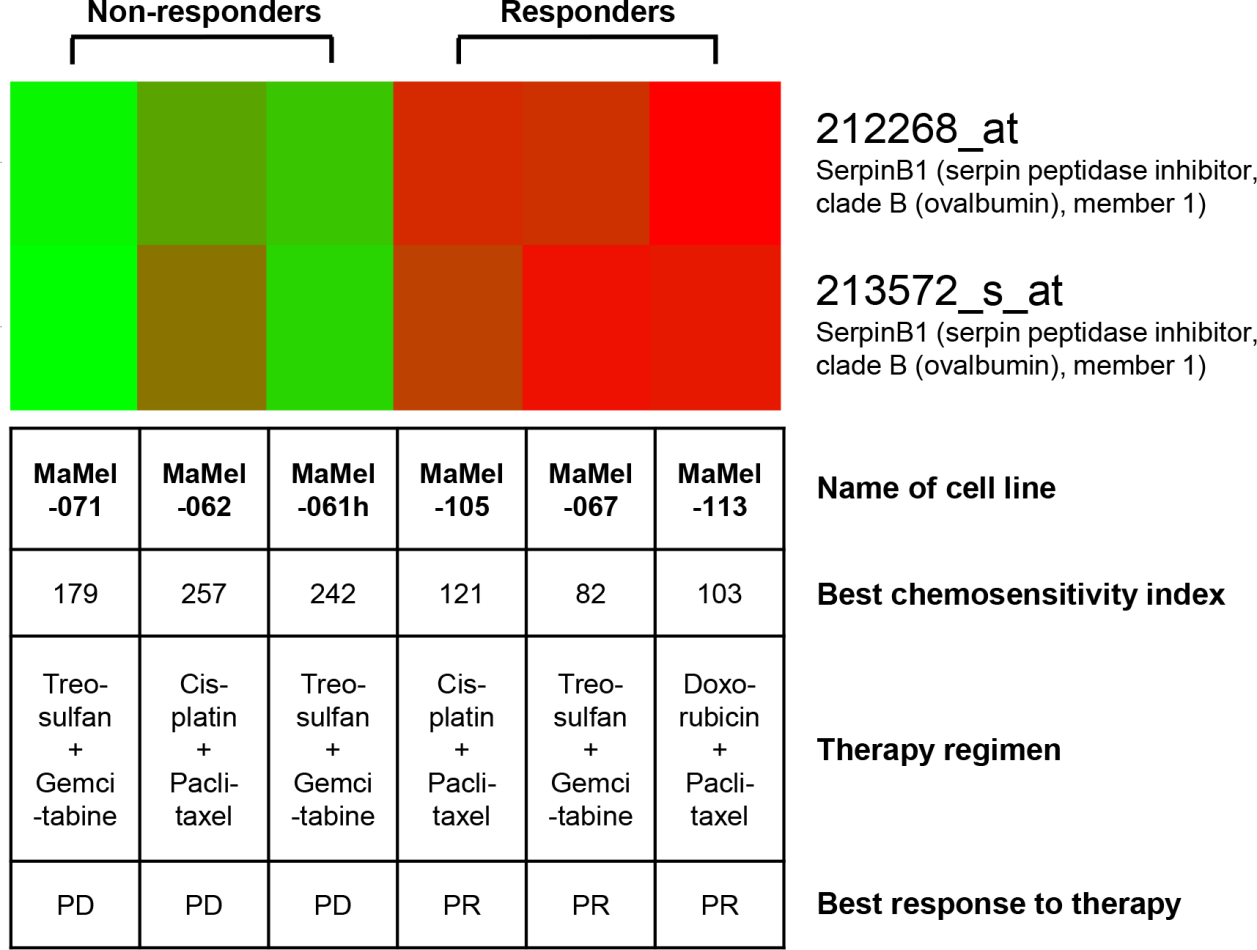
*SERPINB1* gene expression is upregulated in melanoma cell lines derived from clinical responders to chemotherapy as compared to cell lines derived from non-responders The heat map for *SERPINB1* gene expression shows up-regulated gene expression in red, down-regulated gene expression in green. Cell lines were established from metastatic lesions of six melanoma patients biopsied for chemosensitivity testing and analyzed by gene expression profiling using the Affymetrix microarray technology. The best chemosensitivity indices reflect the results of *in vitro* chemosensitivity testing of tumor tissue biospy material also used for the establishment of the cell lines analyzed. The sensivity-directed chemotherapy regimen applied to the corresponding patients after *in vitro* testing as well as its' clinical best response is provided. PR, partial response; PD, progressive disease.

Tumor cell lines established from metastatic lesions of six melanoma patients biopsied for chemosensitivity testing were analyzed by gene expression profiling using the Affymetrix microarray technology. Three cell lines (MaMel-067, MaMel-105, MaMel-113) originated from tumor lesions which presented a clinical response (PR), and three (MaMel-061h, MaMel-062, MaMel-071) were from lesions not responding (PD) to sensitivity-directed chemotherapy (Figure 2). Also, the responders showed lower values for best CSI, reflecting a higher *in vitro* chemosensitivity, than the non-responders (Figure 2). Gene expression profiling revealed 42 genes as more than two-fold up-regulated (Table 2) and 76 genes as more than two-fold down-regulated in melanoma cell lines derived from responders compared to non-responders (Supplementary Table 1). Five candidate genes, *lysyl oxidase-like 1* (*LOXL1*), *secernin 1* (*SCRN1*), *vesicle-associated membrane protein 5* (*VAMP5*), *serine protease inhibitor clade B member 1* (*SERPINB1*), and *thymosin beta 4 X-linked* (*TMSB4X*) were chosen from the list of up-regulated genes by their extent of expression difference and their potential function in chemosensivity/chemoresistance (Table 2). The gene expression data of *SERPINB1* for each tested cell line is depicted in Figure 2.

**Table 2:** Up-regulated genes in chemotherapy responders versus non-responders

Probe Set ID	Gene Title	Gene Symbol	Fold Change
**203570_at**	**lysyl oxidase-like 1**	***LOXL1***	**50, 5**
**201462_at**	**secernin 1**	***SCRN1***	**18, 9**
205483_s_at	interferon, alpha-inducible protein (clone IFI-15K)	*G1P2*	12, 9
**216438_s_at**	**thymosin, beta 4, X-linked** /// thymosin-like 3	***TMSB4X*** /// TMSL3	**11**, 9
212253_x_at	dystonin	*DST*	11, 8
**204929_s_at**	**vesicle-associated membrane protein 5 (myobrevin)**	***VAMP5***	**10, 0**
**212268_at**	**serpin peptidase inhibitor, clade B (ovalbumin), member 1**	***SERPINB1***	**9, 6**
209140_x_at	major histocompatibility complex, class I, B	*HLA-B*	9, 4
209969_s_at	signal transducer and activator of transcription 1, 91kDa	*STAT1*	8, 9
208812_x_at	major histocompatibility complex, class I, C	*HLA-C*	8, 8
209356_x_at	EGF-containing fibulin-like extracellular matrix protein 2	*EFEMP2*	8, 5
214459_x_at	major histocompatibility complex, class I, C	*HLA-C*	8, 0
209124_at	myeloid differentiation primary response gene (88)	*MYD88*	6, 0
207057_at	solute carrier family 16 (monocarboxylic acid transporters), member 7	*SLC16A7*	5, 4
206580_s_at	EGF-containing fibulin-like extracellular matrix protein 2	*EFEMP2*	4, 8
212358_at	CLIP-170-related protein	*CLIPR-59*	4, 7
203595_s_at	interferon-induced protein with tetratricopeptide repeats 5	*IFIT5*	4, 7
203596_s_at	interferon-induced protein with tetratricopeptide repeats 5	*IFIT5*	4, 6
221816_s_at	PHD finger protein 11	*PHF11*	4, 4
219691_at	sterile alpha motif domain containing 9	*SAMD9*	4, 1
209310_s_at	caspase 4, apoptosis-related cysteine peptidase	*CASP4*	4, 1
200887_s_at	signal transducer and activator of transcription 1, 91kDa	*STAT1*	4, 0
202307_s_at	transporter 1, ATP-binding cassette, sub-family B (MDR/TAP)	*TAP1*	3, 9
221840_at	protein tyrosine phosphatase, receptor type, E	*PTPRE*	3, 9
218980_at	formin homology 2 domain containing 3	*FHOD3*	3, 9
201150_s_at	TIMP metallopeptidase inhibitor 3 (Sorsby fundus dystrophy, pseudoinflammatory)	*TIMP3*	3, 9
212254_s_at	dystonin	*DST*	3, 9
203882_at	interferon-stimulated transcription factor 3, gamma 48kDa	*ISGF3G*	3, 8
218986_s_at	hypothetical protein FLJ20035	*FLJ20035*	3, 8
215016_x_at	dystonin	*DST*	3, 6
201649_at	ubiquitin-conjugating enzyme E2L 6	*UBE2L6*	3, 5
210807_s_at	solute carrier family 16 (monocarboxylic acid transporters), member 7	*SLC16A7*	3, 3
202863_at	nuclear antigen Sp100	*SP100*	3, 2
202180_s_at	major vault protein	*MVP*	3, 1
218959_at	homeo box C10	*HOXC10*	3, 1
202771_at	family with sequence similarity 38, member A	*FAM38A*	3, 0
205756_s_at	coagulation factor VIII, procoagulant component (hemophilia A)	*F8*	3, 0
222316_at	Vesicle docking protein p115	*VDP*	2, 8
218373_at	fused toes homolog (mouse)	*FTS*	2, 8
217892_s_at	epithelial protein lost in neoplasm beta	*EPLIN*	2, 8
209398_at	histone 1, H1c	*HIST1H1C*	2, 7
202378_s_at	leptin receptor overlapping transcript	*LEPROT*	2, 7
204062_s_at	unc-51-like kinase 2 (C. elegans)	*ULK2*	2, 7
56256_at	SID1 transmembrane family, member 2	*SIDT2*	2, 6
218309_at	calcium/calmodulin-dependent protein kinase II inhibitor 1	*CAMK2N1*	2, 5
203688_at	polycystic kidney disease 2 (autosomal dominant)	*PKD2*	2, 4
219561_at	coatomer protein complex, subunit zeta 2	*COPZ2*	2, 3
202377_at	leptin receptor /// leptin receptor overlapping transcript	*LEPR //*/ LEPROT	2, 2

Note: Differential gene expression was quantified by Affymetrix microarray analysis of three melanoma cell lines derived from tissue biopsies from responders to chemotherapy as compared to three tumor cell lines derived from non-responders. Differentially expressed genes are sorted by fold change; only genes of > 2fold change are presented. The five candidate genes chosen for further experimental validation are shown in bold letters.

### *SERPINB1* gene expression correlates with *in vitro* sensitivity to cisplatin-containing chemotherapy

qPCR quantification of the relative expression of the five candidate genes *LOXL1*, *SCRN1*, *VAMP5*, *SERPINB1*, and *TMSB4X* in cryopreserved tumor tissues from validation set 1 was correlated to the CSIs measured in the corresponding fresh tissue samples of the same tumor lesions. This analysis revealed that *SERPINB1* expression was associated with the *in vitro* chemosensitivity to cisplatin (*p* = 0.028; *N* = 82), vindesine (*p* = 0.019; *N* = 82), cisplatin + paclitaxel (*p* = 0.0033; *N* = 127), and cisplatin + gemcitabine (*p* = 0.033; *N* = 82). *SERPINB1* expression was not correlated to the *in vitro* chemosensitivity to doxorubicin, paclitaxel, gemcitabine, treosulfan, gemcitabine + treosulfan, gemcitabine + vindesine, and doxorubicin + paclitaxel (data not shown). Thus, all tested regimens containing cisplatin were significantly associated to *SERPINB1* expression, whereas vindesine showed an association as a monotherapeutic only, and not in combination regimens. The expression of the other tested candidate genes *LOXL1*, *SCRN1*, *VAMP5*, and *TMSB4X* did not correlate to the *in vitro* chemosensitivity to any of the tested drugs.

### *SERPINB1* gene expression predicts clinical outcome of cisplatin-based chemotherapy

**Figure 3:**
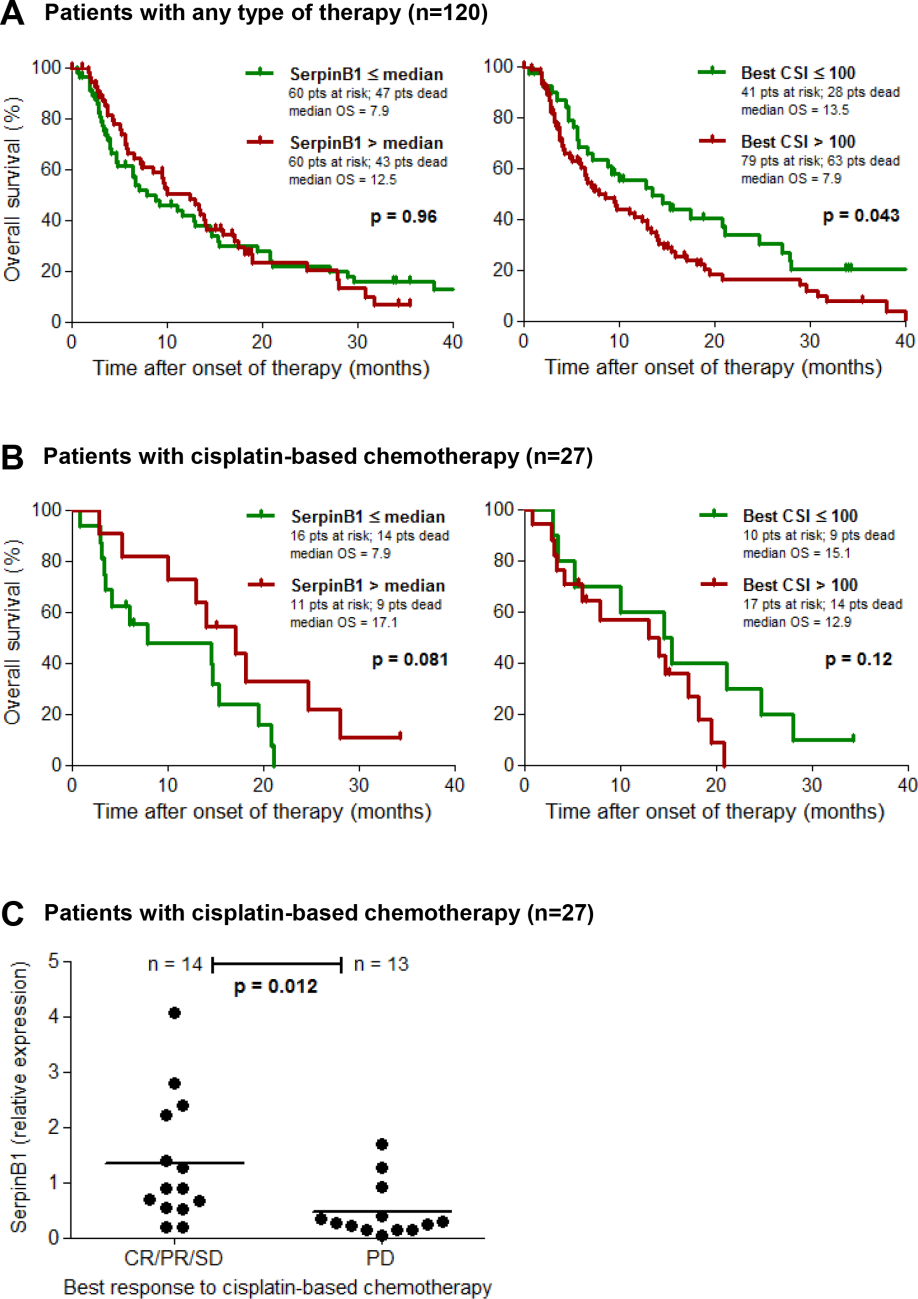
*SERPINB1* gene expression predicts clinical outcome of cisplatin-based chemotherapy Kaplan-Meier plots depicting the probability of overall survival of (**A**) patients from validation set 1 with known clinical follow-up (*n* = 120) including all modes of therapy (chemotherapy, immunotherapy, other/supportive therapy), and (**B**) its subset of patients treated with a cisplatin-based chemotherapy regimen (*n* = 27). Patients are subdivided either by *SERPINB1* relative gene expression in cryopreserved tumor tissues as measured by quantitative real-time PCR, or by the best chemosensitivity index (CSI) of the same tumor lesion determined on fresh tumor tissue by *in vitro* chemosensitivity testing. Differences between groups were calculated using the log rank test. Censored observations are indicated by vertical bars. (**C**) Scatter plot depicting *SERPINB1* relative gene expression, measured as described above, in responders (CR/PR/SD) and non-responders (PD) of the patient subset treated with a cisplatin-based chemotherapy (*n* = 27). Horizontal bars represent means.

The relative expression of *SERPINB1* as measured by qPCR in cryopreserved tumor tissues from validation set 1 was correlated to the patients clinical outcome. Overall survival was first analyzed in all patients with known clinical follow-up data (*n* = 120; see Table 1), comprising all therapy types of the first regimen following chemosensitivity testing (chemotherapy, immunotherapy, other therapy and supportive therapy). In this population we found no association between *SERPINB1* relative expression and survival (*p* = 0.96); however, low values of the best CSI, i.e. CSIs ≤ 100 reflecting a high chemosensitivity, were significantly associated (*p* = 0.043) with a favorable survival in this patient group (Figure 3A). With regard to the subset of patients treated with a chemotherapy regimen containing cisplatin (*n* = 27), patients with high *SERPINB1* expression (> median = 0.88) demonstrated a favorable survival, which did however not reach statistical significance (*p* = 0.081; Figure 3B). The best CSI showed no significant impact on survival in this subgroup (*p* = 0.12; Figure 3B). Multivariate Cox analysis of overall survival in this patient subset revealed serum LDH as the only independent predictor (*p* = 0.016; HR = 11.28; 95%-CI = 1.56–81.32), followed by CSI cisplatin + paclitaxel (*p* = 0.21; HR = 0.37; 95%-CI = 0.08–1.74), gender (*p* = 0.38; HR = 0.56; 95%-CI = 0.15–2.05), *SERPINB1* relative expression (*p* = 0.49; HR = 0.69; 95%-CI = 0.24–1.97), ECOG performance status (*p* = 0.75; HR = 1.27; 95%-CI = 0.29–5.63), and M category (0.99; HR = 1.00; 95%-CI = 0.29–3.42). Tumor response to therapy was grouped as responders (CR/PR/SD) and non-responders (PD). In the subset of patients treated with a cisplatin-based chemotherapy, responders showed significantly higher *SERPINB1* relative expression levels than non-responders (*p* = 0.012; Figure 3C). In the total patient population, there was no significant association of *SERPINB1* expression with therapy response (data not shown).

### SERPINB1 protein expression correlates with *in vitro* sensitivity and clinical outcome of cisplatin-based chemotherapy

**Figure 4:**
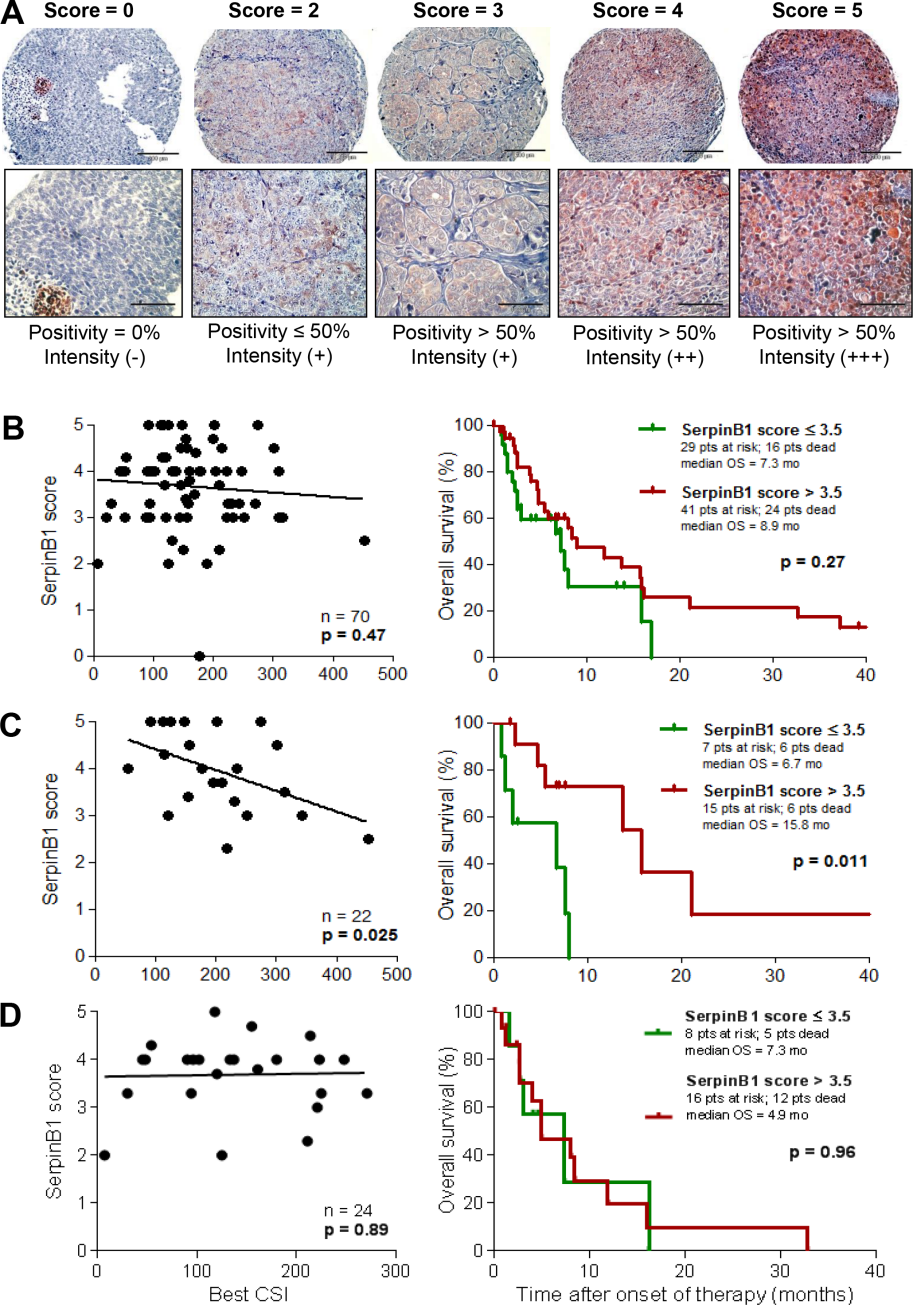
SERPINB1 protein expression in melanoma tissues correlates with *in vitro* and *in vivo* sensitivity to cisplatin-based chemotherapy (**A**) SERPINB1 protein expression as detected by immunohistochemistry on tissue microarrays built from FFPE tissue samples. Representative tissue cores are shown for each value of the expression score. The score is defined as described in the Patients and Methods section. Magnification is 20x (upper row) and 40x (lower row), respectively. SERPINB1 protein expression in tumor tissue samples from patients of validation set 2 (*n* = 70; **B**), and its subsets of patients treated with cisplatin-based chemotherapy (*n* = 22; **C**) or DTIC monochemotherapy (*n* = 24; **D**). Left: Correlation of the SERPINB1 expression score with chemosensitivity indices (CSIs) from *in vitro* sensitivity testing by linear regression analysis. Right: Kaplan-Meier plots depicting the probability of overall survival subdivided by SERPINB1 expression score. Differences between groups were calculated using the log rank test. Censored observations are indicated by vertical bars.

To validate SERPINB1 as a predictor of the outcome of cisplatin-containing chemotherapy, an independent set of patients and respective tumor samples was selected (validation set 2; *n* = 70; see Table 1). In these samples, SERPINB1 expression was detected on protein level by immunohistochemistry in tissue microarrays and quantified by an expression score (Figure 4A). In the whole patient population (*n* = 70) the SERPINB1 expression score had no significant impact on survival (*p* = 0.27; Figure 4B), whereas in the subset of patients treated with a cisplatin-based chemotherapy regimen (*n* = 22), high expression scores of SERPINB1 were associated with a favorable survival (*p* = 0.011; Figure 4C). In contrast, the subset of patients treated with DTIC monochemotherapy revealed no difference in survival when stratified based on SERPINB1 protein expression (*p* = 0.96; Figure 4D). Multivariate analysis of overall survival in this patient subset revealed SERPINB1 expression score (*p* = 0.008; HR = 0.098; 95%-CI = 0.018–0.54) and M category (*p* = 0.048; HR = 19.19; 95%-CI = 1.03–356.77) as independent predictors, followed by serum LDH (*p* = 0.084; HR = 10.96; 95%-CI = 0.73–165.41), gender (*p* = 0.23; HR = 2.89; 95%-CI = 0.52–16.03), CSI cisplatin + paclitaxel (*p* = 0.61; HR = 0.44; 95%-CI = 0.02–10.14), and ECOG performance status (*p* = 0.87; HR = 1.16; 95%-CI = 0.20–6.58). With regard to *in vitro* chemosensitivity, the total patient population showed no correlation between best CSIs and SERPINB1 expression scores (*p* = 0.47; Figure 4B), whereas in patients treated with cisplatin-based regimens low values of the respective CSI correlated with high values of the SERPINB1 expression score (*p* = 0.025; Figure 4C). In patients treated with DTIC, no correlation was found between SERPINB1 expression and chemosensitivity (*p* = 0.89; Figure 4D). Tumor response was not significantly associated with the SERPINB1 expression score in any of the patient subsets.

### Changes in SERPINB1 expression do not functionally impact cisplatin chemosensitivity

**Figure 5:**
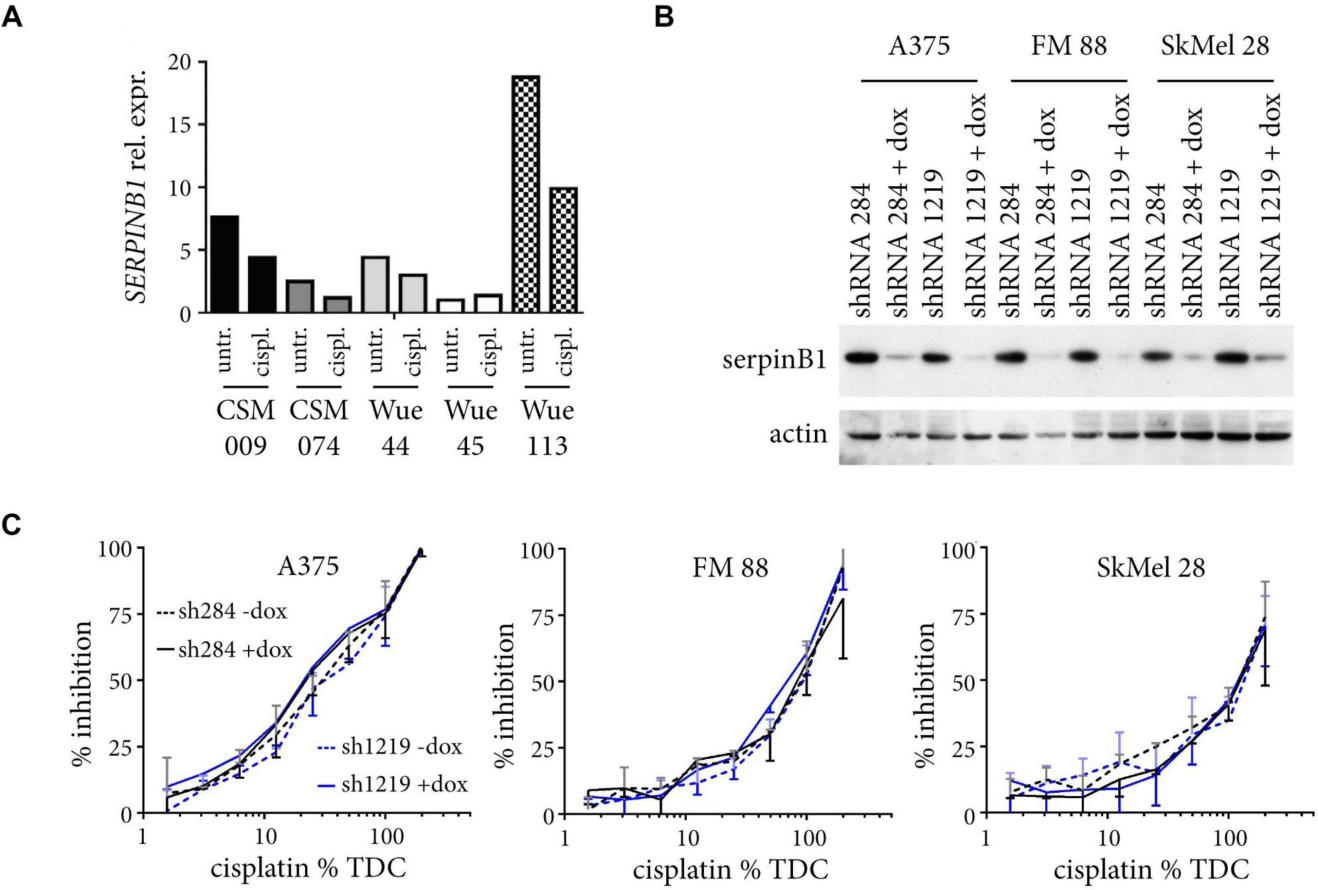
Changes in SERPINB1 expression do not influence cisplatin sensitivity (**A**) Five different melanoma cell lines were cultured for six weeks with increasing doses of cisplatin (6.25%, 12.5%, 25%, 50% TDC). Relative SERPINB1 mRNA expression as quantified by real-time PCR is depicted for cells harvested at the start (untreated; untr.) and the end of the experiment (cisplatin treated; cispl.). (**B**) Melanoma cells infected with SERPINB1-specific shRNA inducible vectors were cultured with or without doxycyclin for four days, and their lysates analyzed for SERPINB1 expression by immunoblotting. ACTIN served as loading control. (**C**) Melanoma cells with or without doxycycline(dox)-induced SERPINB1 knockdown by two different SERPINB1-specific shRNAs were treated for three day with different amounts of cisplatin (6.25% to 200% TDC) and analyzed by the MTS assay. Depicted is the percent inhibition for the given drug concentrations (%TDC) compared to control cells cultivated in normal medium. Given are means from two independent experiments with SD (upper orientation, without dox; lower orientation, with dox).

Our analyses revealed a correlation between SERPINB1 expression and both *in vitro* sensitivity to and clinical outcome of cisplatin-based chemotherapy. Hence, we tested whether this correlation could also be observed experimentally. To this end, five melanoma cells lines with different baseline expression of SERPINB1 were rendered more chemoresistant by cultivating them for six weeks with increasing amounts of cisplatin. SERPINB1 mRNA expression was determined before and after these long-term cisplatin cell culture experiments. In four of the five cell lines the prolonged culture with increasing doses of cisplatin resulted in reduced SERPINB1 expression levels (Figure 5A). Only in one cell line with the lowest SERPINB1 baseline level (WueMel-45), a slight increase in SERPINB1 expression was observed after long-term culture with cisplatin. Notably, prognostic and predictive markers might either be merely associated or influence prognosis or outcome directly by their function for the cells. Consequently, we determined the effect of SERPINB1 expression on cisplatin sensitivity in melanoma cells. First, we transduced three melanoma cell lines with two different inducible *SERPINB1*-specific shRNA vectors. Upon addition of doxycyclin, SERPINB1 expression was almost completely abolished in all cell lines (Figure 5B). However, when sensitivity to cisplatin was measured by MTS assays, no obvious difference could be observed between control and SERPINB1 knockdown cells (Figure 5C).

## DISCUSSION

Predictive markers of chemotherapy outcome in melanoma are rare, and most of those available are analyzed in animal or cell line models only with no regard to clinical data or patient materials. Several serological markers like LDH, S100B, different cytokines, pro-angiogenic factors, and many other molecules are known to be associated with chemotherapy outcome. However, these markers in the first instance are prognostic and not predictive, and therefore are of no help to select patients for specific treatment strategies, or to choose the optimal chemotherapy drugs and regimens. Out of this panel of prognostic serum markers, the most widely investigated one is LDH. Melanoma patients with LDH serum levels beyond normal values are associated with an unfavorable overall survival, and also with non-response and poor progression-free survival under different regimens of chemotherapy [10–13]. However, other therapy strategies like immunotherapy or targeted agents have also been shown to reveal poor response rates and shortened survival times in patients with elevated serum LDH at treatment start [13–15]. In our present study, the patients' serum LDH was measured before treatment onset as a routine for prognostic reasons. As expected, we found a strong association of elevated LDH levels with an impaired survival, with no association to type or specific regimen of therapy (Supplementary Figure 1). Thus, the LDH serum level does not help to select for therapy strategies or to individualize chemotherapy in melanoma.

Tumor tissue-based driver mutations in genes like *BRAF* or *MITF* might be of high impact for the treatment outcome of targeted therapies, but do not correlate with chemotherapy outcome [16, 17]. Other tumor tissue-based molecular markers have been analyzed for their predictive impact for chemotherapy outcome in melanoma in preclinical models only [18, 19]. Studies of molecular markers analyzed in biomaterials from melanoma patients including correlations to clinical chemotherapy response and survival are rare. Hatch and coworkers just described the expression of the endonuclease *XPF-ERCC1* to be associated with the outcome of oxaliplatin chemotherapy in melanoma [20]. In melanoma cell lines, the authors demonstrated that high XPF and ERCC1 protein levels correlate with low sensitivity to oxaliplatin. However, in tumor tissue samples from melanoma patients, the authors found no association between XPF protein expression and clinical response to platin-based therapy regimens. Another recent study demonstrated the *O6-methylguanine-DNA-methyltransferase* (*MGMT*) promoter methylation status determined by PCR in tumor tissue from primary melanomas as a predictive marker of response to temozolomide, an oral derivative of DTIC [21]. Moreover, low MGMT protein expression levels were associated with response to temozolomide. In 2010, Parker and coworkers analyzed FFPE tissues from melanoma patients for a panel of 93 genes associated with chemoresistance by qPCR array technology, and correlated their findings with the *in vitro* chemosensitivity measured by the same methodology as in our present study (ATP-TCA) on fresh tissue samples of the corresponding patients [22]. They found *HSP70*, *EGFR*, and several genes involved in apoptosis, DNA repair, and cell proliferation as common genes associated to chemoresistance towards different cytotoxic agents. *SERPINB1* was not identified as associated to chemoresistance in this study.

SERPINB1 is a member of the large family of serine proteinase inhibitors, the serpins, which also exhibit functions unrelated to inhibition of catalytic activity, such as transport and other mechanisms [23]. SERPINB1 is located intracellularly, and is ubiquitously expressed. As an inhibitor of the neutrophil elastase, it was formerly called monocyte neutrophil elastase inhibitor. SERPINB1 is functional in inflammation and complement activation [24], and has been shown to be involved in neutrophil and megakaryocyte development, as well as in the inhibition of the cytotoxic granule protease granzyme B [23].

The role and specific function of SERPINB1 in cancer biology is largely unknown. SERPINB1 protein has recently been identified by gel electrophoresis and subsequent mass spectrometry to be differentially expressed in gastric and lung carcinoma, respectively, as compared to healthy tissues [25, 26]. Two studies described SERPINB1 as a dose-dependent potent suppressor of metastasis in terms of invasion and migration in oral squamous cell carcinoma, and in lung and breast cancer, respectively [27, 28]. In these cancer entities, it has been shown that *SERPINB* family members, particularly *SERPINB1*, –*B5*, and –*B7*, are differentially expressed in tumor tissues compared to matched normal tissues from the same patients [28]. An overexpression of each of these three genes effectively suppressed the invasiveness and motility of cancer cells. Interestingly, this inhibitory effect was further enhanced by co-expression of any two of them.

Up to now, SERPINB1 expression and function has not been attributed to chemosensitivity and chemotherapy outcome. However, the expression of another member of the serpin family, SERPINB3, together with a second proteinase inhibitor cystatin C, was demonstrated as independent predictors of response to platin-based chemotherapy in NSCLC [29]. Herein, the authors suggest a role of SERPINB3 in the regulation of lysosomal protease-mediated cell death. Indeed, SERPINB3 has been shown to be a negative regulator of programmed cell death in tumor cell lines in response to cytotoxic drugs and ionising radiation [23]. In the current study, however, knockdown of *SERPINB1* did not impact chemosensitivity suggesting that some other factor responsible for chemosensitivity influences SERPINB1 expression.

Taken together, we demonstrated in the present study that gene expression profiling from melanoma tissue is a useful tool to identify differentially expressed genes distinguishing chemosensitive from chemoresistant tumors. From five identified candidate genes, only one revealed a strong correlation to *in vitro* chemosensitivity and clinical chemotherapy outcome by experimental validation in two independent sample sets. Interestingly, the predictive association to *in vitro* and *in vivo* chemosensitivity could be confirmed for cisplatin-containing regimens only. The protein expression of this candidate gene, SERPINB1, proved as a strong and independent predictor of survival after cisplatin-based chemotherapy. Interestingly, all other chemotherapy regimens analyzed, as well as other therapy strategies like immunotherapy, showed no association to SERPINB1 expression. Thus, our results clearly show, that SERPINB1 expression in tumor tissue is not prognostic, like the majority of the already tested potentially predictive markers, but predictive only. This advantage emphasizes SERPINB1 as a useful marker predicting the outcome of cisplatin-based chemotherapies, and may help to personalize chemotherapy of melanoma. Notably, melanoma cell lines under long-term treatment with increasing doses of cisplatin revealed a reduction of SERPINB1 expression. However, knockdown of SERPINB1 expression by shRNA did not influence cisplatin sensitivity in melanoma cell lines.

In conclusion, patients showing strong SERPINB1 protein expression in tumor tissue are likely to benefit from cisplatin-containing chemotherapy regimens; *vice versa*, a low tissue protein expression of this marker would suggest the corresponding patient to be spared a cisplatin-based chemotherapy due to the low probability of response. To confirm the feasibility of SERPINB1 as a biomarker for the personalization of melanoma chemotherapy, these findings should be validated within prospective clinical trials.

## MATERIALS AND METHODS

### Patients and biomaterials

The tumor samples analyzed in this study were collected from patients with histologically confirmed metastatic melanoma, who were biopsied for the purpose of *in vitro* chemosensitivity testing on fresh tumor tissue. Tissue biopsies were taken from metastatic lesions. The native tumor tissue was cleared from connective and fatty tissues and subsequently subjected to chemosensitivity testing. Residual parts of this tumor tissue were used for either cryopreservation, histopathology, or cell culture. Staging of the patients' disease was done according to the American Joint Committee on Cancer (AJCC) classification of 2009 [30]. Patient and tumor characteristics at the time of chemosensitivity testing were extracted from the patients' files. Also, data of the first therapy regimen after *in vitro* testing and its outcome were documented. Therapy regimens were categorized into chemotherapies (all regimens containing at least one chemotherapeutic), immunotherapies (vaccinations, IFN-alpha, IL-2), and other/supportive therapies (sorafenib, doxycyclin, tamoxifen, pamidronate, supportive therapeutics). Individual chemotherapy regimens were selected either sensitivity-directed or by physicians choice. Tumor response was assessed by CT and/or MRI imaging and evaluated according to RECIST [31]. Best response was defined as the best response recorded from start of treatment until disease progression. Overall survival (OS) of the patients was determined from treatment onset until death; otherwise the date of last patient contact was used as the endpoint of survival assessment (censored observation). Collection of biomaterials as well as documentation of clinical data were performed after patients' informed consent and with Institutional Review Board approval (Würzburg 82/07 and 123/08).

### Chemosensitivity testing

Chemosensitivity testing was performed using a non-clonogenic ATP-based luminescence assay (ATP-TCA, DCS Innovative Diagnostic Systems, Hamburg, Germany) as previously described [9]. Briefly, the freshly obtained tumor tissues were minced, enzymatically dissociated to single-cell suspensions, and depleted of red blood cells and debris by Ficoll-Hypaque density gradient centrifugation. This cell suspensions were given into polypropylene round-bottom 96-well plates (2 × 10^4^ cells/well) with or without different chemotherapeutic agents at increasing dilutions (6.25%, 12.5%, 25%, 50%, 100%, 200%) of individual test drug concentrations (TDC), each tested in triplicates. The drugs and TDCs used were cisplatin 3.8 μg/ml, doxorubicin 0.5 μg/ml, vindesine 0.5 μg/ml, paclitaxel 13.6 μg/ml, gemcitabine 12.5 μg/ml, and treosulfan 20 μg/ml, tested individually or in combinations. After seven days of incubation at 37°C, 5% CO_2_ and 100% humidity, the cells were lysed and the ATP content of the lysate was quantified by a luciferin-luciferase luminescence reaction using a microplate luminometer (Berthold Detection Systems, Pforzheim, Germany). Cell suspensions incubated without chemotherapeutic agents were used as control. Chemosensitivity indices (CSIs) ranging from 0 to 600 were calculated for each test drug or drug combination by summing up the percentages of cell viability at the six drug concentrations tested [32]. Thus, a CSI of 600 indicates full cell viability/minimal drug sensitivity, whereas a CSI of 0 reflects complete cell death/maximal drug sensitivity. Best CSI was defined as the lowest CSI of all drugs and combinations tested for a tumor sample.

### Cell culture and gene expression profiling

Permanently growing melanoma cell lines were established from residual tissue specimen derived from biopsies taken for chemosensitivity testing. These as well as the established melanoma cell lines A375, FM 88, and SKMel 28, were maintained in RPMI 1640 (Life Technologies, Grand Island, NY) supplemented with 10% fetal calf serum (Life Technologies), 5 mM L-glutamine, 100 U/ml penicillin and 100 μg/ml streptomycin at 37°C in a humidified 5% CO_2_ atmosphere. The obtained cell lines were used for analysis after at least six culture passages. Cell lines were chosen for gene expression profiling by Affymetrix chip technology by the clinical response of the corresponding patients to sensitivity-directed chemotherapy. Total RNA was isolated from 2 × 10^6^ cells with commercially available purification kits (Gentra Systems, Minneapolis, MN), and thereafter subjected to a second clean-up by a silica-gel-based membrane using RNeasy Mini Kit (Qiagen, Hilden, Germany). Concentrations of DNA and RNA were measured by UV spectrophotometry and OD 260/280 nm ratios between 1.9 and 2.1 were obtained for all RNA samples. 400 ng of total RNA isolated from each cell line was used to check for integrity on Bioanalyzer 2100 System (Agilent Technologies, Palo Alto, CA). Sample preparation and hybridisation was done as described before [33]. Thereafter, samples were loaded on Human HG-U133A 2.0 micro-arrays (Affymetrix, Sunnydale, CA) comprising 22,277 sequences. Image analysis and pair-wise comparison of expression profiles between cell lines from responders and non-responders were performed with the Affymetrix GeneChip Operating Software (GCOS) as described before [33].

### Quantitative real-time PCR

Five genes showing significant expression differences between responders and non-responders in micro-array analysis were assessed on mRNA level by quantitative real-time PCR (qPCR). For RNA isolation from 1.5 × 10^6^ cells or 25–50 20 μm sections of cryopreserved tissues the PeqGOLD Total RNA kit (Peqlab, Erlangen, Germany) was used according to the manufacturer's instructions. Subsequently, cDNA was synthesized from 1 μg total RNA using the Superscript II Reverse Transcriptase cDNA Kit (Invitrogen, Carlsbad, CA). For real-time PCR the Absolute qPCR Low ROX Mix (Thermo Fisher Scientific, Waltham, MA) was used following the manufacturer's protocol. A 20 μl reaction contained 1 μl cDNA, 300 nM of forward and reverse primer and 100 nM of the respective dual-labelled probe. The standard thermal profile of the 7500 Fast Real-Time PCR machine (Life Technologies, Darmstadt, Germany) was applied. Primers and probes were designed with locations in different exons using the software Primer Express 3 (Applied Biosystems, Foster City, CA). Primers were: LoxL1_sense TGC CAG TGG ATC GAC ATA ACC, LoxL1_anti-sense CGT TGT TGG TGA AGT CAG ACT C, Vamp5_sense CTC CGC AGG CAG AGA AGC, Vamp5_anti-sense CAT AAT TTC CGT CAC CTC GTT CG, SCRN1_sense TGA TTG TGG ATC GTG ATG AAG C, SCRN1_anti-sense CAT CTT AGT GGT GAG CGA AAG C, SerpinB1_sense TGC ATA TGG CTA CAT CGA GGA C, SerpinB1_anti-sense TCC AAA GTC AAC TGT TCC TCA ATC, TMSB4X_sense CGA AAC TGA AGA AGA CAG AGA CG, TMSB4X_anti-sense GCA CGC CTC ATT ACG ATT CG, RPLP0_sense CCA TCA GCA CCA CAG CCT TC, RPLP0_anti-sense GGC GAC CTG GAA GTC CAA CT; probes were: LoxL1_probe AAC TAC ATC CTC AAG GTG CAC GTG AAC CC, Vamp5_probe TAT TCC TGC CAT CGC TGC TGC CGC, SCRN1_probe AAT GCA CCT CAC TCC CTC TGT GAC TTT CTC, SerpinB1_probe TGC CGT GTG CTG GAA CTG CCT TAC C, TMSB4X_probe TCC ACT GCC TTC CAA AGA AAC GAT TGA ACA, RPLP0_probe ATC TGC TGC ATC TGC TTG GAG CCC A. The expression of each target gene was normalized to that of the housekeeping gene *RPLP0*. Relative expression levels were calculated by the ΔΔCt method using LIVAk KJ as calibrator [34]. Five melanoma cells lines established from biopsies taken for chemosensitivity testing were treated over six weeks with increasing concentrations of Cisplatin (1 μM to 10 μM). RNA isolated from cells at the start and end of the treatment period were subjected to real time PCR to determine relative expression levels of *SERPINB1* calibrated to Wue45 untreated.

### Tissue microarray and immunohistochemistry

For histopathology, representative parts of the tumor tissue biopsies were formalin-fixed, paraffin-embedded (FFPE), and thereafter stained with hematoxylin and eosin (H + E) as well as with melanoma-specific markers (HMB45, Melan-A/MART-1) for diagnosis confirmation. Representative tumor areas were marked on H + E-stained slides. Thereafter, three 0.6 mm punch cores were taken from the corresponding areas on the FFPE blocks and inserted into grids on new paraffin blocks by use of the manual tissue arrayer MTA-1 (Beecher Instruments, Sun Prairie, WI, USA). 5 μm sections were cut from these tissue microarrays (TMAs) and stained for SERPINB1 using specific antibodies (HPA018871, Sigma Aldrich, Munich, Germany; dilution 1:500). Three independent investigators who were blinded to the clinical data examined these slides. SERPINB1 staining intensity was graded as no (−/0), weak (+/1), moderate (++/2), and strong (+++/3) staining. The percentage of SERPINB1-positive cells among all tumor cells of each core was graded as 0% (0), 1 – 50% (1), and 51% – 100% (2) positive cells. The sum of staining intensity and percentage of positive cells was defined as SERPINB1 expression score, and ranged from 0 to 5. For each tumor, an average was calculated from the scores of all tissue cores available.

### SERPINB1 knockdown

Knockdown of SERPINB1 was realized by an inducible lentiviral shRNA vector based on a system previously described [35]. Two different shRNA sequences targeting *SERPINB1* were cloned into the vectors 289: GGA GCG TCT TAT ATT CTG AAC TCG AGT TCA GAA TAT AAG ACG CTA, and 1219: AGT GCT TTA TTA CCT GAG TTC TCG AGA ACT CAG GTA ATA AAG CAC T. Melanoma cells were transduced by lentiviral particles produced in HEK293T [36] and selected by puromycin addition four days after infection. For induction of shRNA expression cells were treated for four days with doxycyclin (1 μg/ml). Knockdown of SERPINB1 was confirmed by immunoblotting with an anti-SERPINB1 antibody (HPA018871, Sigma Aldrich); ACTIN (ab5694; Abcam, Cambridge, UK) served as loading control. The effect of SERPINB1 knockdown on the sensitivity to cisplatin was determined by MTS assays. In brief, melanoma cells transduced with SERPINB-specific shRNA vectors were seeded in 96 well plates. To half of the wells doxycyclin was added. The next day all wells received the appropriate cisplatin concentration, i.e. 0%, 6.25%, 12.5%, 25%, 50%, 100% or 200% TDC. After three days of culture, the metabolic activity was measured as recommended by the manufacturer (Promega, Mannheim, Germany). The percentage of inhibition was calculated as (value_untreated_ – value_treated_)/value_untreated_.

### Statistical analysis

Survival curves and median survival times were calculated using the Kaplan-Meier method for censored failure time data. The log rank test was used for comparison of survival probabilities between groups. The proportional hazards model of Cox was used to identify independent predictors of survival testing candidate markers in adjustment with the relevant clinical covariates gender (male versus female), M category (M1a/b versus M1c), ECOG performance status (0 versus ≥ 1), serum LDH (≤ UNL versus > UNL), and CSI (≤ 100 versus > 100). The proportional-hazard assumption was tested based on Schoenfeld residuals. Linear regression was used to detect correlations between CSIs and marker expressions. Student's *t* test was used to compare groups of therapy response. *P*-values < 0.05 were considered statistically significant and were not corrected for multiple testing.

## SUPPLEMENTARY MATERIALS TABLE AND FIGURE



## References

[1] Menzies AM, Long GV (2013). Recent advances in melanoma systemic therapy. BRAF inhibitors, CTLA4 antibodies and beyond. Eur J Cancer.

[2] Eggermont AM, Spatz A, Robert C (2014). Cutaneous melanoma. Lancet.

[3] Batus M, Waheed S, Ruby C, Petersen L, Bines SD, Kaufman HL (2013). Optimal management of metastatic melanoma: current strategies and future directions. Am J Clin Dermatol.

[4] Espinosa E, Grob JJ, Dummer R, Rutkowski P, Robert C, Gogas H, Kefford R, Eggermont AM, Martin AS, Hauschild A, Schadendorf D (2015). Treatment algorithms in stage IV melanoma. Am J Ther.

[5] Eigentler TK, Caroli UM, Radny P, Garbe C (2003). Palliative therapy of disseminated malignant melanoma: a systematic review of 41 randomised clinical trials. Lancet Oncol.

[6] Korn EL, Liu PY, Lee SJ, Chapman JA, Niedzwiecki D, Suman VJ, Moon J, Sondak VK, Atkins MB, Eisenhauer EA, Parulekar W, Markovic SN, Saxman S (2008). Meta-analysis of phase II cooperative group trials in metastatic stage IV melanoma to determine progression-free and overall survival benchmarks for future phase II trials. J Clin Oncol.

[7] Awada A, Vandone AM, Aftimos P (2012). Personalized management of patients with solid cancers: moving from patient characteristics to tumor biology. Curr Opin Oncol.

[8] Griewank KG, Scolyer RA, Thompson JF, Flaherty KT, Schadendorf D, Murali R (2014). Genetic Alterations and Personalized Medicine in Melanoma: Progress and Future Prospects. J Natl Cancer Inst.

[9] Ugurel S, Schadendorf D, Pfohler C, Neuber K, Thoelke A, Ulrich J, Hauschild A, Spieth K, Kaatz M, Rittgen W, Delorme S, Tilgen W, Reinhold U (2006). *In vitro* drug sensitivity predicts response and survival after individualized sensitivity-directed chemotherapy in metastatic melanoma: a multicenter phase II trial of the Dermatologic Cooperative Oncology Group. Clin Cancer Res.

[10] Agarwala SS, Keilholz U, Gilles E, Bedikian AY, Wu J, Kay R, Stein CA, Itri LM, Suciu S, Eggermont AM (2009). LDH correlation with survival in advanced melanoma from two large, randomised trials (Oblimersen GM301 and EORTC 18951). Eur J Cancer.

[11] Weide B, Elsässer M, Büttner P, Pflugfelder A, Leiter U, Eigentler TK, Bauer J, Witte M, Meier F, Garbe C (2012). Serum markers lactate dehydrogenase and S100B predict independently disease outcome in melanoma patients with distant metastasis. Br J Cancer.

[12] O'Day SJ, Eggermont AM, Chiarion-Sileni V, Kefford R, Grob JJ, Mortier L, Robert C, Schachter J, Testori A, Mackiewicz J, Friedlander P, Garbe C, Ugurel S (2013). Final results of phase III SYMMETRY study: randomized, double-blind trial of elesclomol plus paclitaxel versus paclitaxel alone as treatment for chemotherapy-naive patients with advanced melanoma. J Clin Oncol.

[13] Weide B, Richter S, Büttner P, Leiter U, Forschner A, Bauer J, Held L, Eigentler TK, Meier F, Garbe C (2013). Serum S100B, lactate dehydrogenase and brain metastasis are prognostic factors in patients with distant melanoma metastasis and systemic therapy. PLoS One.

[14] Abusaif S, Jradi Z, Held L, Pflugfelder A, Weide B, Meier F, Garbe C, Eigentler TK (2013). S100B and lactate dehydrogenase as response and progression markers during treatment with vemurafenib in patients with advanced melanoma. Melanoma Res.

[15] Kelderman S, Heemskerk B, van Tinteren H, van den Brom RR, Hospers GA, van den Eertwegh AJ, Kapiteijn EW, de Groot JW, Soetekouw P, Jansen RL, Fiets E, Furness AJ, Renn A (2014). Lactate dehydrogenase as a selection criterion for ipilimumab treatment in metastatic melanoma. Cancer Immunol Immunother.

[16] Ugurel S, Houben R, Schrama D, Voigt H, Zapatka M, Schadendorf D, Bröcker EB, Becker JC (2007). Microphthalmia-associated transcription factor (MITF) gene amplification in metastatic melanoma is a prognostic marker for patient survival, but not a predictive marker for chemosensitivity and chemotherapy response. Clin Cancer Res.

[17] Meckbach D, Keim U, Richter S, Leiter U, Eigentler TK, Bauer J, Pflugfelder A, Buttner P, Garbe C, Weide B (2014). BRAF-V600 mutations have no prognostic impact in stage IV melanoma patients treated with monochemotherapy. PLoS One.

[18] Yue J, Lu H, Liu J, Berwick M, Shen Z (2012). Filamin-A as a marker and target for DNA damage based cancer therapy. DNA Repair (Amst).

[19] Yue J, Lan S, Yuan C, Shen Z (2012). Prognostic values of filamin-A status for topoisomerase II poison chemotherapy. Int J Biol Sci.

[20] Hatch SB, Swift LP, Caporali S, Carter R, Hill EJ, MacGregor TP, D'Atri S, Middleton MR, McHugh PJ, Sharma RA (2014). XPF protein levels determine sensitivity of malignant melanoma cells to oxaliplatin chemotherapy: suitability as a biomarker for patient selection. Int J Cancer.

[21] Schraml P, von Teichman A, Mihic-Probst D, Simcock M, Ochsenbein A, Dummer R, Michielin O, Seifert B, Schlappi M, Moch H, von MR (2012). Predictive value of the MGMT promoter methylation status in metastatic melanoma patients receiving first-line temozolomide plus bevacizumab in the trial SAKK 50/07. Oncol Rep.

[22] Parker KA, Glaysher S, Polak M, Gabriel FG, Johnson P, Knight LA, Poole M, Narayanan A, Hurren J, Cree IA (2010). The molecular basis of the chemosensitivity of metastatic cutaneous melanoma to chemotherapy. J Clin Pathol.

[23] Heit C, Jackson BC, McAndrews M, Wright MW, Thompson DC, Silverman GA, Nebert DW, Vasiliou V (2013). Update of the human and mouse SERPIN gene superfamily. Hum Genomics.

[24] Law RH, Zhang Q, McGowan S, Buckle AM, Silverman GA, Wong W, Rosado CJ, Langendorf CG, Pike RN, Bird PI, Whisstock JC (2006). An overview of the serpin superfamily. Genome Biol.

[25] Liu Y, Li Y, Tan BB, Zhao Q, Fan LQ, Zhang ZD, Li ZX (2013). Technique appraisement of comparative proteomics and screening of differentiation-related protein in gastric carcinoma. Hepatogastroenterology.

[26] Pastor MD, Nogal A, Molina-Pinelo S, Melendez R, Salinas A, Gonzalez DlP, Martin-Juan J, Corral J, Garcia-Carbonero R, Carnero A, Paz-Ares L (2013). Identification of proteomic signatures associated with lung cancer and COPD. J Proteomics.

[27] Tseng MY, Liu SY, Chen HR, Wu YJ, Chiu CC, Chan PT, Chiang WF, Liu YC, Lu CY, Jou YS, Chen JY (2009). Serine protease inhibitor (SERPIN) B1 promotes oral cancer cell motility and is over-expressed in invasive oral squamous cell carcinoma. Oral Oncol.

[28] Chou RH, Wen HC, Liang WG, Lin SC, Yuan HW, Wu CW, Chang WS (2012). Suppression of the invasion and migration of cancer cells by SERPINB family genes and their derived peptides. Oncol Rep.

[29] Petty RD, Kerr KM, Murray GI, Nicolson MC, Rooney PH, Bissett D, Collie-Duguid ES (2006). Tumor transcriptome reveals the predictive and prognostic impact of lysosomal protease inhibitors in non-small-cell lung cancer. J Clin Oncol.

[30] Balch CM, Gershenwald JE, Soong SJ, Thompson JF, Atkins MB, Byrd DR, Buzaid AC, Cochran AJ, Coit DG, Ding S, Eggermont AM, Flaherty KT, Gimotty PA (2009). Final version of 2009 AJCC melanoma staging and classification. J Clin Oncol.

[31] Therasse P, Arbuck SG, Eisenhauer EA, Wanders J, Kaplan RS, Rubinstein L, Verweij J, Van Glabbeke M, van Oosterom AT, Christian MC, Gwyther SG (2000). New guidelines to evaluate the response to treatment in solid tumors. European Organization for Research and Treatment of Cancer, National Cancer Institute of the United States, National Cancer Institute of Canada. J Natl Cancer Inst.

[32] Andreotti PE, Cree IA, Kurbacher CM, Hartmann DM, Linder D, Harel G, Gleiberman I, Caruso PA, Ricks SH, Untch M, Sartori C, Bruckner HW (1995). Chemosensitivity testing of human tumors using a microplate adenosine triphosphate luminescence assay: clinical correlation for cisplatin resistance of ovarian carcinoma. Cancer Res.

[33] Bloethner S, Chen B, Hemminki K, Müller-Berghaus J, Ugurel S, Schadendorf D, Kumar R (2005). Effect of common B-RAF and N-RAS mutations on global gene expression in melanoma cell lines. Carcinogenesis.

[34] Livak KJ, Schmittgen TD (2001). Analysis of relative gene expression data using real-time quantitative PCR and the 2(−Delta Delta C(T)) Method. Methods.

[35] Herold MJ, van den Brandt J, Seibler J, Reichardt HM (2008). Inducible and reversible gene silencing by stable integration of an shRNA-encoding lentivirus in transgenic rats. Proc Natl Acad Sci U S A.

[36] Houben R, Adam C, Baeurle A, Hesbacher S, Grimm J, Angermeyer S, Henzel K, Hauser S, Elling R, Brocker EB, Gaubatz S, Becker JC, Schrama D (2012). An intact retinoblastoma protein-binding site in Merkel cell polyomavirus large T antigen is required for promoting growth of Merkel cell carcinoma cells. Int J Cancer.

