# Supplementary material for: SERPINB1 expression is predictive for sensitivity and outcome of cisplatin-based chemotherapy in melanoma
Source: Oncotarget. 2016 Jan 20;7(9):10117–32. doi: 10.18632/oncotarget.6956 (PMC4891108; doi:10.18632/oncotarget.6956)
Supplement: Supplementary file 1 [file oncotarget-07-10117-s001.pdf]

SERPINB1 expression is predictive for sensitivity and outcome of cisplatin-based chemotherapy in melanoma

Supplementary Materials

Supplementary Table S1: Down-regulated genes in chemotherapy responders versus non-responders

Probe set ID	Full name of gene	Gene symbol	Fold change
205694_at	tyrosinase-related protein 1	*TYRP1*	54, 2
213712_at	elongation of very long chain fatty acids-like 2	*ELOVL2*	25, 3
219263_at	ring finger protein 128	*RNF128*	21, 1
206786_at	histatin 3 / cingulin-like 1	*HTN3/CGNL1*	17, 5
219159_s_at	SLAM family member 7	*SLAMF7*	17, 2
211470_s_at	sulfotransferase family, cytosolic, 1C, member 1	*SULT1C1*	16, 6
206893_at	sal-like 1 (Drosophila)	*SALL1*	13, 5
205342_s_at	sulfotransferase family, cytosolic, 1C, member 1	*SULT1C1*	12, 4
220116_at	potassium intermediate/conductance calcium-activated channel, subfamily N, member 2	*KCNN2*	10, 0
203680_at	protein kinase, cAMP-dependent, regulatory, type II, beta	*PRKAR2B*	9, 1
210372_s_at	tumor protein D52-like 1	*TPD52L1*	8, 3
222361_at	Tubulin, beta 8	*TUBB8*	7, 6
218816_at	leucine rich repeat containing 1	*LRRC1*	7, 2
204947_at	E2F transcription factor 1	*E2F1*	6, 7
220234_at	carbonic anhydrase VIII	*CA8*	6, 1
202094_at	baculoviral IAP repeat-containing 5 (survivin)	*BIRC5*	5, 3
210145_at	phospholipase A2, group IVA (cytosolic, calcium-dependent)	*PLA2G4A*	5, 2
220954_s_at	paired immunoglobin-like type 2 receptor beta	*PILRB*	5, 1
201998_at	ST6 beta-galactosamide alpha-2,6-sialyltranferase 1	*ST6GAL1*	5, 0
202870_s_at	CDC20 cell division cycle 20 homolog (S. cerevisiae)	*CDC20*	4, 9
209621_s_at	PDZ and LIM domain 3	*PDLIM3*	4, 8
212094_at	paternally expressed 10	*PEG10*	4, 8
202743_at	phosphoinositide-3-kinase, regulatory subunit 3 (p55, gamma)	*PIK3R3*	4, 7
219217_at	hypothetical protein FLJ23441	*FLJ23441*	4, 7
209569_x_at	DNA segment on chromosome 4, 234 expressed sequence	*D4S234E*	4, 6
221436_s_at	cell division cycle associated 3	*CDCA3*	4, 6
208089_s_at	tudor domain containing 3	*TDRD3*	4, 5
210983_s_at	MCM7 minichromosome maintenance deficient 7	*MCM7*	4, 4
220615_s_at	male sterility domain containing 1	*MLSTD1*	4, 2
203775_at	solute carrier family 25, member 13 (citrin)	*SLC25A13*	4, 2
216860_s_at	growth differentiation factor 11	*GDF11*	4, 1
210233_at	interleukin 1 receptor accessory protein	*IL1RAP*	4, 1
211732_x_at	histamine N-methyltransferase	*HNMT*	4, 1
219148_at	PDZ binding kinase	*PBK*	4, 0

202095_s_at	baculoviral IAP repeat-containing 5 (survivin)	*BIRC5*	3, 9
209172_s_at	centromere protein F, 350/400ka (mitosin)	*CENPF*	3, 8
202580_x_at	forkhead box M1	*FOXM1*	3, 8
210334_x_at	baculoviral IAP repeat-containing 5 (survivin)	*BIRC5*	3, 8
203755_at	BUB1 budding uninhibited by benzimidazoles 1 homolog beta	*BUB1B*	3, 8
218281_at	mitochondrial ribosomal protein L48	*MRPL48*	3, 7
205048_s_at	phosphoserine phosphatase	*PSPH*	3, 7
219763_at	DENN/MADD domain containing 1A	*DENND1A*	3, 7
203510_at	met proto-oncogene (hepatocyte growth factor receptor)	*MET*	3, 7
203358_s_at	enhancer of zeste homolog 2 (Drosophila)	*EZH2*	3, 6
219372_at	carnitine deficiency-associated, expressed in ventricle 1	*CDV1*	3, 6
204822_at	TTK protein kinase	*TTK*	3, 5
202589_at	thymidylate synthetase	*TYMS*	3, 4
219978_s_at	nucleolar and spindle associated protein 1	*NUSAP1*	3, 4
201890_at	ribonucleotide reductase M2 polypeptide	*RRM2*	3, 4
214581_x_at	tumor necrosis factor receptor superfamily, member 21	*TNFRSF21*	3, 4
210074_at	cathepsin L2	*CTSL2*	3, 3
219537_x_at	delta-like 3 (Drosophila)	*DLL3*	3, 3
202954_at	ubiquitin-conjugating enzyme E2C	*UBE2C*	3, 3
201291_s_at	topoisomerase (DNA) II alpha 170kDa	*TOP2A*	3, 3
218663_at	chromosome condensation protein G	*HCAP-G*	3,3
210999_s_at	growth factor receptor-bound protein 10	*GRB10*	3, 2
204170_s_at	CDC28 protein kinase regulatory subunit 2	*CKS2*	3, 2
218755_at	kinesin family member 20A	*KIF20A*	3, 1
210052_s_at	TPX2, microtubule-associated, homolog (Xenopus laevis)	*TPX2*	3, 1
205761_s_at	dihydrouridine synthase 4-like (S. cerevisiae)	*DUS4L*	3, 0
208079_s_at	serine/threonine kinase 6	*STK6*	3, 0
210720_s_at	amyloid beta (A4) precursor protein-binding, family A	*APBA2BP*	2, 9
32502_at	glycerophosphodiester phosphodiesterase domain containing 5	*GDPD5*	2, 9
201259_s_at	synaptophysin-like 1	*SYPL1*	2, 9
201774_s_at	chromosome condensation-related SMC-associated protein 1	*CNAP1*	2, 8
201292_at	topoisomerase (DNA) II alpha 170kDa	*TOP2A*	2, 7
204112_s_at	histamine N-methyltransferase	*HNMT*	2, 7
203764_at	discs, large homolog 7 (Drosophila)	*DLG7*	2, 7
201271_s_at	RNA binding protein, autoantigenic	*RALY*	2, 7
204318_s_at	G-2 and S-phase expressed 1	*GTSE1*	2, 7
200790_at	ornithine decarboxylase 1	*ODC1*	2, 6
207828_s_at	centromere protein F, 350/400ka (mitosin)	*CENPF*	2, 5
222077_s_at	Rac GTPase activating protein 1	*RACGAP1*	2, 5
203564_at	Fanconi anemia, complementation group G	*FANCG*	2, 4
219119_at	LSM8 homolog, U6 small nuclear RNA associated	*LSM8*	2, 4
203554_x_at	pituitary tumor-transforming 1	*PTTG1*	2, 3

Differential gene expression was quantified by Affymetrix microarray analysis of three melanoma cell lines derived from tissue biopsies from responders to chemotherapy as compared to three tumor cell lines derived from non-responders. Differentially expressed genes are sorted by fold change; only genes of > 2fold change are presented.

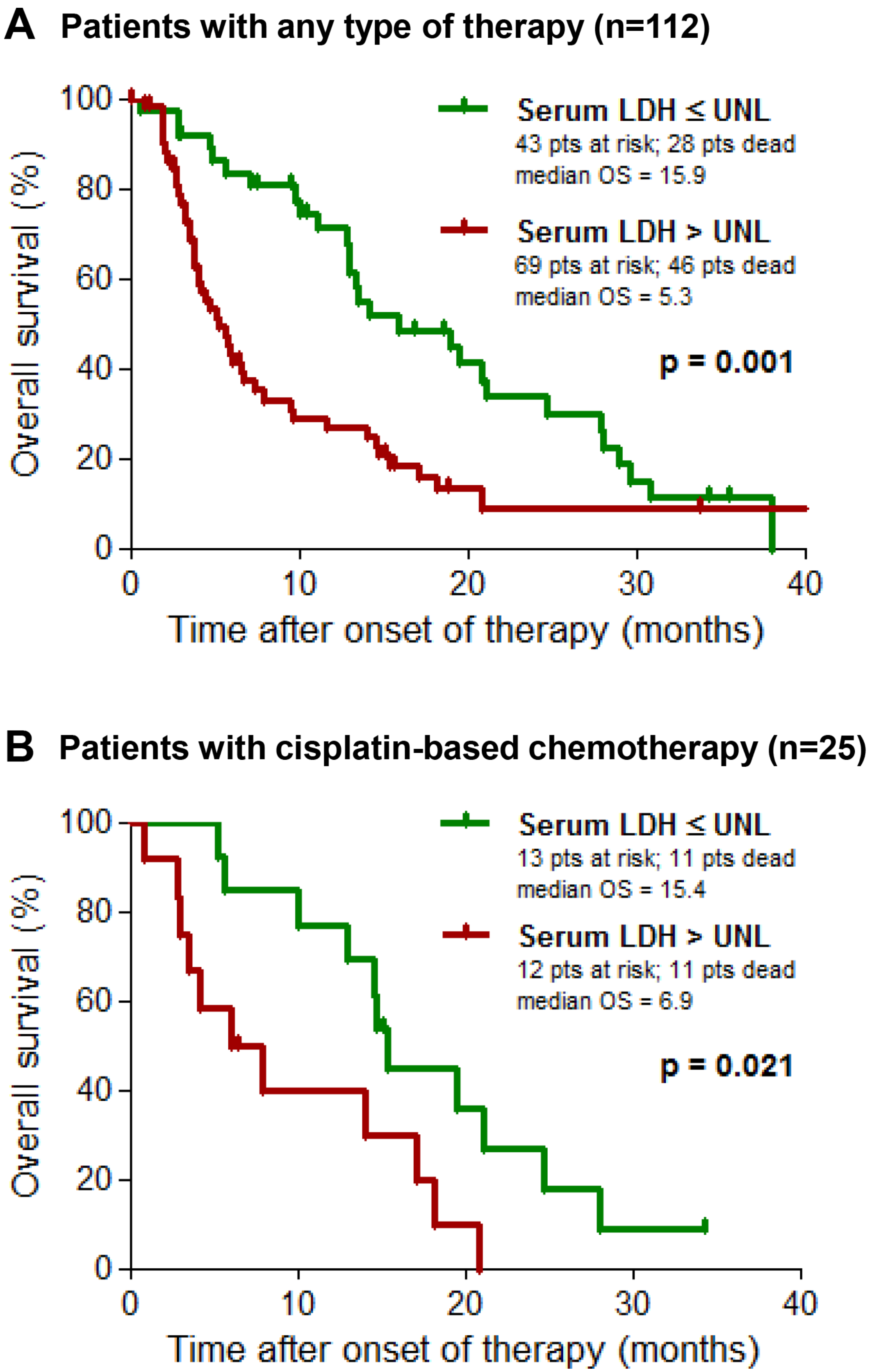

Supplementary Figure S1: Serum LDH as a prognostic marker in metastatic melanoma independent of the mode of therapy. Kaplan Meier plots depicting the probability of overall survival of (**A**) patients of validation set 1 (*n* = 112; LDH serum values missing for 15 patients) including all types of therapy (chemotherapy, immunotherapy, other/supportive therapy), and (**B**) its subset of patients treated with a cisplatin-based chemotherapy regimen (*n* = 25; LDH serum values missing for 2 patients). Differences between groups were calculated using the log rank test. Censored observations are indicated by vertical bars.
